# Comparative profiling of the transcriptional response to iron restriction in six serotypes of *Actinobacillus pleuropneumoniae *with different virulence potential

**DOI:** 10.1186/1471-2164-11-698

**Published:** 2010-12-09

**Authors:** Kirstine Klitgaard, Carsten Friis, Øystein Angen, Mette Boye

**Affiliations:** 1 National Veterinary Institute, Technical University of Denmark, Bülowsvej 27, DK-1790, Copenhagen, Denmark; 2 National Food Institute, Technical University of Denmark, Kemitorvet, building 204, DK-2800 Kgs. Lyngby, Denmark

## Abstract

**Background:**

Comparative analysis of gene expression among serotypes within a species can provide valuable information on important differences between related genomes. For the pig lung pathogen *Actinobacillus pleuropneumoniae*, 15 serotypes with a considerable variation in virulence potential and immunogenicity have been identified. This serotypic diversity can only partly be explained by amount of capsule and differences in the RTX toxin genes in their genomes. Iron acquisition *in vivo *is an important bacterial function and in pathogenic bacteria, iron-limitation is often a signal for the induction of virulence genes. We used a pan-genomic microarray to study the transcriptional response to iron restriction *in vitro *in six serotypes of *A. pleuropneumoniae *(1, 2, 3, 5b, 6, and 7), representing at least two levels of virulence.

**Results:**

In total, 45 genes were significantly (p < 0.0001) up-regulated and 67 genes significantly down-regulated in response to iron limitation. Not previously observed in *A. pleuropneumoniae *was the up-regulation of a putative *cirA*-like siderophore in all six serotypes. Three genes, recently described in *A. pleuropneumoniae *as possibly coding for haemoglobin-haptoglobin binding proteins, displayed significant serotype related up-regulation to iron limitation. For all three genes, the expression appeared at its lowest in serotype 3, which is generally considered one of the least virulent serotypes of *A. pleuropneumoniae*. The three genes share homology with the *hmbR *haemoglobin receptor of *Neisseria meningitidis*, a possible virulence factor which contributes to bacterial survival in rats.

**Conclusions:**

By comparative analysis of gene expression among 6 different serotypes of *A. pleuropneumoniae *we identified a common set of presumably essential core genes, involved in iron regulation. The results support and expand previous observations concerning the identification of new potential iron acquisition systems in *A. pleuropneumoniae*, showing that this bacterium has evolved several strategies for scavenging the limited iron resources of the host. The combined effect of iron-depletion and serotype proved to be modest, indicating that serotypes of both moderate and high virulence at least *in vitro *are reacting almost identical to iron restriction. One notable exception, however, is the haemoglobin-haptoglobin binding protein cluster which merits further investigation.

## Background

*Actinobacillus pleuropneumoniae*, is a Gram-negative, facultative anaerobic coccobacillus of the *Pasteurellaceae *family [1]. It is the causative agent of porcine pleuropneumonia. This highly infectious disease causes impaired animal welfare and serious economic losses in the swine industry, world-wide. The infection can lead to both peracute disease with rapid death and chronic infection resulting in asymptomatic carriers [2]. Based on differences in capsular polysaccharides, 15 serotypes have been recognized [3]. The serotypes differ greatly in both virulence potential, immunogenicity and in geographical distribution [4-8]. Due to differences in immunogenicity, vaccines raised against one serotype do not provide protection from infection by other serotypes [8].

A number of virulence factors have been described for *A. pleuropneumoniae *[2,9-11]. Serotype variations in virulence potential seem to be primarily governed by the amount of capsule and the combination of RTX toxins, denoted *apxI*, *apxII*, and *apxIII*, produced by the individual serotypes [12,13]. The most virulent combination, *apxI *and *apxII*, is produced by serotypes 1, 5, 9, and 11. *ApxII *and *apxIII *are found in the medium virulent serotypes 2, 3, 4, 6, 8, and 15. The remaining serotypes produce one toxin: *apxII *by serotypes 7, 12, and 13 and *apxI *by serotypes 10, and 14 [12]. Serotypes 7 and 12 are also considered to be of medium virulence, while serotypes 10, 13 and 14 are only rarely isolated from disease [4,14]. Still, observations of variation in pathogenic potential, even among serotypes and strains expressing the same *apx *toxins, indicate that other virulence determinants must be contributing to the observed differences in pathogenesis [2,15-17]. Serotype 3 is generally believed to be less virulent than the remaining types [4,18], although some serotype 3 strains showed no difference in pathogenicity when compared to other *apxII*/*apxIII *producing serotypes [7,17].

An important virulence factor for bacteria is the ability to survive and grow in an iron-limited environment [2]. Iron is involved in metabolic pathways, respiration, oxygen transport, DNA synthesis and synthesis of metabolites [19,20] and is critical to the invading microorganisms for establishing infection. As part of the innate defense, the mammalian host keeps the levels of intracellular free iron to around 10^-18^M which is insufficient to allow bacterial growth [19]. The low level of free iron in the host is maintained by high affinity proteins such as transferrin, lactoferrin, haem, haemoglobin (Hb), and ferritin [19].

Like other pathogens, *A. pleuropneumoniae *has adapted a number of strategies for scavenging host iron. The bacterium is able to use porcine transferrin as well as haem proteins as sole sources of iron. *A. pleuropneumoniae *genes known to be involved in iron uptake are the porcine transferrin specific outer membrane (OM) proteins, *tbpA *and *tbpB*, the co-transcribed *tonB-exbB*-*exbD *complex [21-23], and the second *tonB *system, designated *exbB2*-*exbD2*-*tonB2 *[24]. Solely responsible for the Hb uptake in *A. pleuropneumoniae *is the haemoglobin binding protein, *hgbA *[25]. The presence of a periplasmic binding protein-dependent transport system, homologuous to *yfeABCD *of *Yersinia pestis *has been documented in *A. pleuropneumoniae *and other *Pasteurellaceae *species [26-29]. A gene cluster sharing homology with the *HmbR *Hb receptor from *N. meningitides *has recently been identified by microarray analysis [26]. In *A. pleuropneumoniae*, the putative *Actinobacillus *ferric uptake operon, *afuABC*, and the siderophore ferrichrome uptake, *fhu*, receptor are not regulated by iron-levels [30-33].

In many bacteria the intracellular iron level and utilization is controlled by the balance between the regulatory protein, *fur *(ferric-uptake regulator protein), and ryhB [20,34]. *Fur *is a global gene regulator involved in numerous functions of the cell, such as respiration, glycolysis, purine metabolism, and redox-stress resistance [20]. It represses transcription upon interaction with its co-repressor Fe^2+^, and causes de-repression in the absence of Fe^2+ ^[19]. The *fur *regulated ryhB, a small non-coding RNA (sRNA), acts by repressing iron-using proteins under iron-restricted conditions [20]. Whether ryhB or other sRNAs are involved in the regulatory response of *Pasteurellaceae *remains to be demonstrated. Recently, however, homologues of the global sRNA regulator, Hfq, a key factor in regulations by sRNAs in bacteria, has also been identified in *A. pleuropneumoniae*, *P. multocida*, and *H. influenza *[35]. Among the many Hfq-dependent regulators is ryhB [35].

Microarray analysis of gene regulation under iron restriction have been studied in *A. pleuropneumoniae*, *Pasteurella multocida*, *Haemophilus influenza, Mannheimia haemolytica*, and *Haemophilus parasuis*, respectively [26-29,36]. Presently, however, the knowledge of intra-species variation in response to iron deprivation is limited for the *Pasteurellaceae *family. Only few comparative transcriptional profiling studies have been performed in this group and none for *A. pleuropneumoniae *[37]. We used an *in vitro *model system to compare the response of moderate (serotypes 2, 3, 6, 7), and highly virulent strains (serotypes 1 and 5) of *A. pleuropneumoniae *to the iron restricted conditions found in the porcine host. The primary aims were: 1) to identify any variations in the transcriptional response among the serotypes which might contribute to the explanation of the observed differences in virulence, 2) to identify a set of genes defining the core modulon of *A. pleuropneumoniae *in response to iron limitation, 3) to develop a valid method for transcriptional comparison of multiple serotypes.

## Results & Discussion

### Microarray analysis of *A. pleuropneumoniae *iron regulation

**Figure 1:**
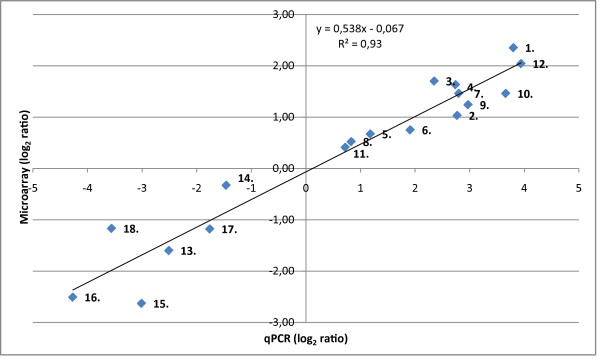
**qPCR validation of microarray results**. Mean log_2 _ratios of qPCR results, based on all the included serotypes, are plotted against the mean log_2 _ratios of the microarray analysis. Included are twelve up-regulated and six down-regulated genes representing a range of log_2 _ratio values. The numbers on the graph refer to genes listed in Table 4.

Based on all the sequence information available for *A. pleuropneumoniae*, we designed a pan-genomic microarray, targeting the *A. pleuropneumoniae *serotypes 1, 2, 3, 5, 6, and 7, respectively. This array was used to study the gene expression of all the above mentioned serotypes in response to iron limitation. The study set-up was established in a previous investigation [38]. Briefly, gene expression of bacteria grown for 30 min in the presence of 300 μM of the iron chelator 2,2'-dipyridyl was compared to control cultures. The length of the iron limitation period was chosen on the basis of previous results, where expression of *tbpA *and *exbB *appeared to reach maximum level 30 min after the addition of an iron chelator [38]. Still, a longer iron starvation period may have revealed more genes to be significantly altered by iron deprivation. For each serotype, the growth experiment was performed in four replicas on different days. Real-time quantitative RT-PCR (qPCR) was used to verify the microarray results on a sub-set of 18 up- or down-regulated genes, representing a wide range of log_2 _ratio values (Figure 1). Three previously validated reference genes were used for normalization of the qPCR data [38]. Of the two methods, qPCR displayed the greatest dynamic range. Still, a correlation of 0.93 between microarray and qPCR log_2 _expression ratios (Spearman's Rho, p < 3.51 × 10^-8^, df = 16) demonstrated that the results of the two platforms correlated very well with each other. Additionally, cDNA from samples of serotype 2 and 6 grown in iron replete media (2,2'-dipyridyl and ammonium iron(II) sulfate hexahydrate) were included in the qPCR analysis. Comparing the bacterial expression in iron replete versus iron deplete media (only 2,2'-dipyridyl added), nearly all the tested genes were oppositely or much less up- or down-regulated in the iron replete media of both serotypes (Additional file 1 Figure S1). Only one gene, *copA*, was up-regulated in both iron deplete and replete cells of serotype 6. Based on the results of the qPCR analysis, along with previous observation by other researchers using the same iron chelator [27,29], we concluded that most of the observed differences in the microarrays were actually due to iron deprivation and not the addition of 2,2'-dipyridyl.

**Figure 2:**
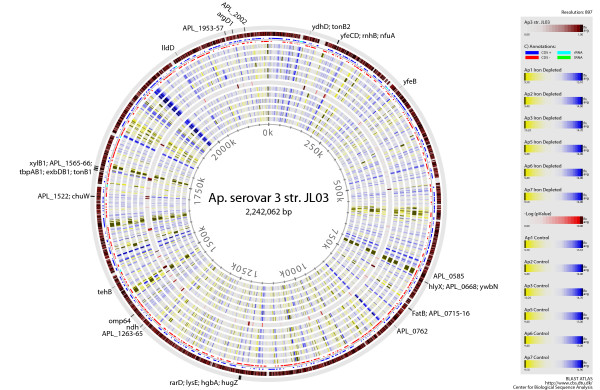
**Genome BLAST atlases of *A. pleuropneumoniae *expression patterns**. *A. pleuropneumoniae *3 str. JL03 is the reference genome and is compared to the remaining five serotypes, including controls (K) (rings 1 to 6 from center) and iron depleted cultures (JH) (rings 8 to 13 from center). The p values of the expression differences between control and iron depleted cultures are included (ring 7 from centre). The positions of the ORFs displaying significant up-regulation under iron restriction are marked.

**Figure 3:**
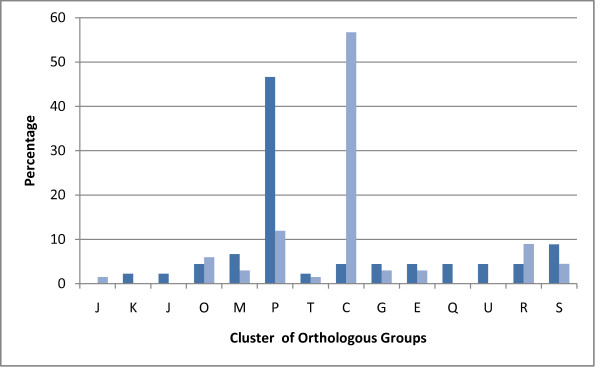
**Distribution of significantly regulated genes classified according to the Clusters of Orthologous groups (COGs)**. Dark blue bars: up-regulated genes, light blue bars: down-regulated genes. J: translation, ribosomal structure and biogenesis, K: transcription, L: DNA replication, recombination, and repair, O: posttranslational modification, protein turnover, chaperones, M: cell envelope biogenesis, outer membrane, P: inorganic ion transport and metabolism, T: signal transduction mechanisms, C: energy production and conversion, G: carbohydrate transport and metaholism, E: amino acid transport and metabolism, Q: secondary metabolites biosynthesis, transport, and catabolism, U: intracellular trafficking, secretion, and vesicular transport, R: general function prediction, only, S: function unknown.

Of the 4876 target genes on the microarray, a total of 112 genes were found to be significantly expressed (p < 0.0001; estimated false positive error rate of 0.5%) in all six serotypes under iron depleted conditions. Of these, 45 were up-regulated (Table 1) and 67 were down-regulated (Table 2). When estimating the combined effect of treatment and serotype, 12 genes also displayed significant (p < 0.0001; estimated false positive error rate of 0.5%) serotype related response to iron limitation, three were up-regulated and 9 down-regulated (marked in Table 1 and 2). A quick overview of the general distribution of the significantly regulated genes is provided in the genomic atlases depicting the gene expression of all the included serotypes (Figure 2). When classifying the significantly regulated genes according to the Clusters of Orthologous Groups (COGs, http://www.ncbi.nlm.nih.gov/COG, [39]), there was a striking difference in the distribution of functional groups between the up- and down-regulated genes (Figure 3). Nearly half (47%) the up-regulated genes belonged to the "Inorganic ion transport and metabolism" category while this group of proteins only represented 12% of the down-regulated genes. The down-regulated genes mainly (57%) belonged to the "Energy production and conversion" category, a group which only constituted 4.5% of the up-regulated genes. In general, we observed a pattern of gene expression very similar to previous observations of transcriptional response to iron limitation in *Pasteurellaceae*: an increased uptake of iron via the mobilization of iron-transport genes and siderophores and a decrease in iron consumption by down-regulation of non-essential iron consuming proteins, mostly genes involved in anaerobic metabolism [26-29].

**Table 1:** Genes up-regulated in A. pleuropneumoniae under iron limitation

Locus tag*	Gene	Gene function	COGs**	Ratio (log2)
APL_2002	-	hypothetical protein	P	0.75
APL_1974	*argD1*	diaminobutyrate-2-oxoglutarate aminotransferase	E	0.20
APL_1957^+^	-	Lipoprotein_5 domain containing protein	S	3.36
APL_1956^+^	-	Putative N-methylhydantoinase B/acetone carboxylase, alpha subunit	S	2.74
APL_1955^+^	*hpuB^#^*	Probable hemoglobin-haptoglobin-binding protein 2 precursor	P	1.63
APL_1954^+^	*hpuB^#^*	Hemoglobin-haptoglobin utilization protein B precusor	P	1.70
APL_1953^+^	*hpuB^#^*	Hemoglobin-haptoglobin-binding protein A precursor	P	1.03
APL_1849^+^	*lldD*	L-lactate dehydrogenase [cytochrome]	C	2.04
APL_1806	-	hypothetical protein	S	0.98
APL_1571^+^	*tonB1*	Periplasmic protein	M	1.46
APL_1570^+^	*exbB1*	Biopolymer transport protein	U	2.35
APL_1569^+^	*exbD1*	Biopolymer transport protein	U	1.77
APL_1568^+^	*tbpB1*	Transferrin-binding protein 2 precursor	P	0.92
APL_1567^+^	*tbpA1*	Transferrin-binding protein 1; tonB dependent receptor	P	1.24
APL_1566	-	Hypothetical protein	S	1.40
APL_1565	-	Putative gluconolactonase (Glucose secondary pathway)	G	1.42
APL_1564^+^	*xylB1*	Sugar (Pentulose and hexulose) kinase	G	1.20
APL_1523^+^	*chuW*	Probable oxygen-independent coproporphyrinogen III oxidase	H	0.62
APL_1522	-	Predicted nucleoside-diphosphate-sugarepimerase	MG	0.88
APL_1350^+^	*tehB*	Tellurite resistance protein	P	0.73
APL_1299^+^	*hemR*	Iron-regulated OM protein; tonB dependent receptor	P	1.19
APL_1271^+^	*ndh*	NADH dehydrogenase (part of oxidative phosphorylation pathway)	C	0.37
APL_1265	*copA*	Copper-transporting P-type ATPase	P	1.46
APL_1264	-	Cation transport ATPase	P	1.81
APL_1263	-	Predicted metal-binding protein	R	1.60
APL_1048^+^	*hugZ*	Putative heme utilization protein	P	2.41
APL_1047^+^	*hgbA*	Probable haemoglobin-and-haptoglobin binding protein 4	P	1.81
APL_1046	*lysE*	Lysine exporter protein	E	1.84
APL_1045^+^	*rarD*	Predicted permease	R	0.66
APL_0762	-	SAM-dependent methyltransferase	Q	0.29
APL_0716	-	Iron(III) ABC transporter, permease protein	P	0.40
APL_0715	-	Iron(III) transport system permease protein	P	0.43
APL_0714^+^	*FatB*	ABC-type enterochelin transport system, periplasmic component	P	0.36
APL_0669^+^	*ywbN*	Putative iron dependent peroxidase	P	0.75
APL_0668^+^	-	Predicted periplasmic lipoprotein involved in iron transport	P	0.66
APL_0656^+^	*hlyX*	Fumarate/nitrate reduction transcriptional regulator	T	0.52
APL_0585	-	AcrR protein, putative HTH-type transcriptional regulator	K	0.29
APL_0565	*cirA*	Hypothetical ABC transporter ATP-binding protein	P	0.67
APL_0271^+^	*yfeB*	Iron (chelated) transporter, ATP-binding protein	P	0.76
APL_0149	*nfuA*	Fe/S biogenesis protein	O	0.62
APL_0129	*rnhB*	RNase HII; binds manganese; endonuclease which specifically degrades the RNA of RNA-DNA hybrids (DNA synthesis pathway)	L	0.50
APL_0128^+^	*yfeC*	Putative iron transport system membrane protein	P	0.45
APL_0127^+^	*yfeD*	Iron (chelated) transport system membrane protein	P	0.42
APL_0076^+^	*tonB2*	Protein tonB	M	0.41
(APL_0073	*ydhD*	Conserved monothiol glutaredoxin-like protein	O	0.47

* Organized according to Locus tag numbers of *A. pleuropneumoniae *serotype 5 in GenBank.

** Clusters of Orthologous Groups.

^+ ^Genes in common with *A. pleuropneumoniae *serotype 1 [26].

^# ^Genes significantly differentially expressed among serotypes.

**Table 2:** Genes down-regulated in A. pleuropneumoniae under iron limitation

Locus tag	Gene	Gene function	COG**	Ratio (log2)
APL_1757^+^	*fumC*	fumarate hydratase	C	-0.83
APL_1675	*dms B*	anaerobic dimethyl sulfoxide reductase chain B	C	-2.00
APL_1674^+^	*dmsA*	Anaerobic dimethyl sulfoxide reductase chain A precursor	C	-2.07
APL_1572	-	Predicted membrane protein	S	-0.67
APL_1546	*hcp*	hydroxylamine reductase, catalyzes the reduction of hydroxylamine to ammonia and water (Fe-S cluster containing protein)	C	-1.28
APL_1529^+^	*frdA*	Fumarate reductase flavoprotein subunit	C	-0.90
APL_1528^+^	*frdB*	Fumarate reductase iron-sulfur subunit	C	-0.90
APL_1527^+^	*frdC*	Fumarate reductase subunit C	C	-1.39
APL_1526	*frdD*	Fumarate reductase subunit D	C	-1.37
APL_1496		Predicted esterase	R	-0.24
APL_1432	-	Putative NAD(P)H oxidoreductase	R	-0.66
APL_1431^+^	*napF*	Ferredoxin-type protein	C	-1.23
APL_1430	*napD*	putative periplasmic nitrate reductase protein	P	-1.66
APL_1429	*napA*	Periplasmic nitrate reductase	C	-1.14
APL_1428	*napG*	Quinol dehydrogenase periplasmic component	C	-1.39
APL_1427	*napH*	Quinol dehydrogenase membrane component	C	-1.14
APL_1426	*napB*	Nitrate reductase cytochrome c-type subunit	C	-1.60
APL_1422	*napC*	Nitrate/TMAO reductase	C	-1.42
APL_1379	*mauG^#^*	Cytochrome c peroxidase	P	-1.95
APL_1367^+^	*ccmF*	Cytochrome c-type biogenesis protein	O	-0.54
APL_1337	*hypG*	Hydrogenase maturation factor	O	-0.68
APL_1336	*hybE*	Hydrogenase 2-specific chaperone	S	-1.00
APL_1335	*hyaD*	Ni, Fe-hydrogenase maturation factor	C	-0.99
APL_1334	*hyaB*	Ni, Fe-hydrogenase I large subunit	C	-1.06
APL_1333	*hybB*	Putative cytochrome b subunit of the hydrogenase 2	C	-1.17
APL_1332	*hybA2*	Fe-S-cluster-containing hydrogenase components 1	C	-1.95
APL_1331^+^	*hyaA^#^*	Ni, Fe-hydrogenase I small subunit	C	-2,51
APL_1328	*hybD^#^*	Hydrogenase maturation factor	O	-1.90
APL_1327	*hypB^#^*	Hydrogenase nickel incorporation protein	OK	-2.1
APL_1316^+^	*dcuB2*	Anaerobic C4-dicarboxylate membrane transporter	R	-0.78
APL_1253	-	Di- and tricarboxylate transporter	P	-0.83
APL_1216	*luxS*	S-ribosylhomocysteine lyase	T	-0.39
APL_1213	-	Predicted phosphatase/phosphohexomutase	R	-1.21
APL_1237	-	Possible integral membrane sulfate transporter	P	-0.64
APL_1162	-	Predicted iron-dependent peroxidase	P	-0.41
APL_1156	*citT*	Di-and tricarboxylate transporter	P	-1.20
APL_1129	*cytB562*	Soluble cytochrome b562	C	-0.48
APL_1124^+^	*pfkA*	6-phosphofructokinase	G	-0.37
APL_1086^+^	*ompW*	OM protein ompW precursor	M	-1.53
APL_1036^+^	*pflB*	Formate acetyltransferase	C	-0.38
APL_1011^+^	*adh2*	Alcohol dehydrogenase, class IV/NAD-dependent aldehyde dehydrogenases	C	-1.24
APL_0966	-	Putative transport protein	R	-0.71
APL_0895	*fdnI*	Formate dehydrogenase, gamma subunit	C	-1.15
APL_0894^+^	*fdxH*	Formate dehydrogenase, iron-sulfur subunit	C	-1.17
APL_0893^+^	*fdxG*	Formate dehydrogenase, nitrate-inducible, major subunit	C	-1.01
APL_0892	*fdxG*	Formate dehydrogenase, nitrate-inducible, major subunit	C	-1.18
APL_0857	*sdaA^#^*	L-serine dehydratase (gluconeogenesis)	E	-1.00
APL_0856	*sdaC^#^*	serine transporter	E	-1.58
				
APL_0780	*lrgA2^#^*	Putative effector of murein hydrolase LrgA	R	-1.11
APL_0779	-	Putative effector of murein hydrolase	M	-0.90
APL_0723	*tgt*	Queuine tRNA-ribosyltransferase	J	-0.15
APL_0689^+^	*torY*	Cytochrome c-type protein	C	-2.30
APL_0688^+^	*torZ*	Trimethylamine-N-oxide reductase precursor	C	-1.61
APL_0607^+^	nfnB	Putative NAD(P)H nitroreductase	C	-0.54
APL_0446^+^	ykgE	Putative dehydrogenase subunit, Fe-S oxidoreductase	C	-2.63
APL_0445^+^	ykgF	Iron-sulfur electron transport protein	C	-1.79
APL_0444^+^	engA	Putative GTP binding protein	S	-1.10
APL_0416	-	N-acetyl-D-glucosamine kinase	KG	-0.36
APL_0155^+^	*nqrF*	Na+-translocating NADH-ubiquinone oxidoreductase subunit F	C	-0.37
APL_0154^+^	*nqrE*	Na+-transporting NADH-ubiquinone oxidoreductase subunit E	C	-0.35
APL_0153^+^	*nqrD*	Na+-translocating NADH-ubiquinone oxidoreductase subunit D	C	-0.47
APL_0152^+^	*nqrC*	Na+-translocating NADH-ubiquinone oxidoreductase subunit C	C	-0.33
APL_0151^+^	*nqrB*	Na+-translocating NADH-ubiquinone oxidoreductase subunit B	C	-0.40
APL_0150^+^	*nqrA*	Na+-translocating NADH-ubiquinone oxidoreductase subunit A	C	-0.46
APL_0103	*nrfD^#^*	Nitrate reductase, transmembrane protein	P	-1.51
APL_0101^+^	*nrfB^#^*	Cytochrome c nitrite reductase pentaheme subunit	C	-2.17
APL_0100^+^	*nrfA^#^*	Nitrate reductase, cytochrome c552, catalyzes the formate-dependent reduction of nitrite to ammonia;	P	-2.34

* Organized according to Locus tag numbers of *A. pleuropneumoniae *serotype 5 in GenBank.

** Clusters of Orthologous Groups.

^+ ^Genes in common with *A. pleuropneumoniae *serotype 1 [26].

^# ^Genes significantly differentially expressed among serotypes.

### Genes up-regulated under growth in iron-depleted media

Not surprisingly, many of the up-regulated genes in this study were related to iron homeostasis (Table 1); at least 60% of these were previously identified in *A. pleuropneumoniae *serotype 1 under similar conditions by Deslandes et al. [26]. Some genes were homologous to iron-regulators commonly found in the *Pasteurellaceae *group or in Gram-negative bacteria, and known to be of major importance to iron sequestration of the host. Among these were the *tonB1*-*exbB1*-*exbD1 *complex, universally found in Gram-negative bacteria [25,40], and the two co-regulated transferrin receptor genes *tbpB1 *and *tbpA1 *[21,23]. *TonB2*, but not *exbB2*-*exbD2*, was up-regulated. In accordance with previous observations, the up-regulation of *tonB2 *was lower than in *tonB1 *(log2 ratios of 0.41 and 1.46, respectively) [24]. Also highly expressed under iron restriction were OM Hb receptor *hgbA *and the putative haem utilization gene, *hugZ *[25,26]. As in most transcriptional profiling studies of *Pasteurellaceae *species under iron deprivation, members of the *yfe *system likewise showed up-regulated transcription [26-29].

#### Putative hemoglobin and haem uptake genes

As previously observed in *A. pleuropneumoniae *serotype 1, iron depletion resulted in up-regulation of a putative *tonB*-dependent haem receptor (APL_1299) [26]. APL_1299 encodes a protein with significant similarity to *tdhA*/*hemR*/*huxC *genes of other *Pasteurellaceae *species [41]. This gene appears to be important for heme-hemopexin uptake during host adaptation in *H. influenzae *and *P. multocida *[42]. APL_1299 is probably of less significance in *A. pleuropneumoniae*, where the *hgbA *gene appears to be sufficient for heme/iron acquisition from the mammal host [25]. A more interesting virulence candidate is the tellurite resistance gene, *tehB*. *TehB *increased its expression during growth in iron restrictive media in all six serotypes of *A. pleuropneumoniae*, as well as in *P. multocida *and in *H. influenzae *[26,27]. In *H. influenzae*, *tehB *is involved in both resistance to oxidative damage and haem uptake/utilization, and is an important virulence factor in this organism in an animal model of invasive disease [43]. Further studies are needed to determine whether *tehB *is also of importance to iron-sequestration in *A. pleuropneumoniae*.

**Figure 4:**
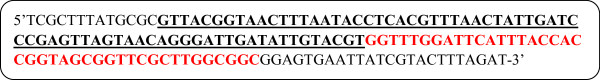
**Variable region of ORF APL_1953 illustrated in serotype 7**. Red text: region with high GC concentration (57% in serotype 7). Underscored text: region missing in *A. pleuropneumoniae *serotypes 2, 5, and 6.

A new potential iron acquisition system identified by Deslandes et al. [26] was also up-regulated under iron limited conditions in this study. The three putative open reading frames (ORFs), APL_1953 to APL_1955, all displayed between 50% to 55% homology with the *tonB*-dependent haemoglobin-haptoglobin receptor, *hpuB*, of *N. meningitidis *[44]. In *N. meningitidis*, slip-strand mispairing is the cause of phase variation in the expression of *hpuAB *[44]. In the ORF APL_1953 of *A. pleuropneumoniae*, we observed serotype related sequence variation in the form of a 71 bp insertion/deletion region, situated just before a 42 bp sequence of high GC content (62% GC in serotype 5 against a normal average of 41%) (Figure 4). Such GC rich sequences can be regions of high recombination [45]. In serotypes 2, 5, and 6, the 71 bp were missing, whereas the sequence was present in serotypes 1, 3, and 7. We PCR amplified and sequenced the part of APL_1953 harboring the variable region in all six serotypes. This confirmed that the observed differences were not due to assembly error in the published genomes. In the short version, APL_1953 codes for a putative protein of 19.7 kDa and in the long version a 26.7 kDa sized protein. Judging from our results, APL_1953 appeared to be expressed in all the included *A. pleuropneumoniae *serotypes. Such an insertion/deletion event may have an important role in transitions between commensalism and pathogenicity [45]. In this case, both variants of the APL_1953 gene included a high-virulent serotype. Consequently, it is doubtful if this particular region is related to virulence. Still, it would be interesting to examine the possible effect of the insertion/deletion region with regard to protein function.

**Figure 5:**
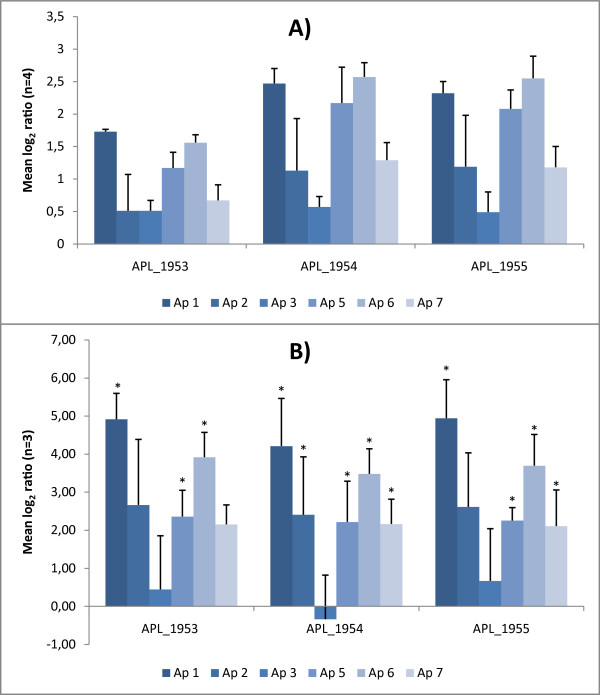
**Serotype specific transcriptional up-regulation in response to iron depletion**. Individual mean log_2 _expression ratios for the three *hpuB *ORFs, APL_1953 to APL_1955 in the six included *A. pleuropneumoniae *serotypes: (A) obtained with microarray analysis, (B) obtained with qPCR analysis. Hinges represent the 95% confidence intervals. The asterisks indicate statistical significant up-regulation (P < 0.05) as determined by simple statistical randomization test in REST [71].

The three *hpuB *ORFs also displayed serotype-specific transcriptional variations in response to iron levels of the media. Here, the most intriguing observation was the low expression of all three genes in serotype 3, which, is considered one of the least virulent *A. pleuropneumoniae *serotypes (Figure 5A) [4,18]. In the qPCR analysis, serotype 3 was the only serotype in which none of the three ORFs were significantly up-regulated during iron deprivation (Figure 5B). For the remaining serotypes, we observed no correlation between level of pathogenesis and degree of expression. Serotype 2, for example, was only significantly up-regulated in APL_1954 and in the moderately virulent serotype 6, all three genes were highly up-regulated. As previously mentioned, infection experiments have shown that differences in pathogenic potential also exists within serotypes [17]. Therefore comparisons of strains of the same serotype which have experimentally been determined to be of differential virulence might elucidate the role of these ORFs in *A. pleuropneumoniae*.

Srikumar et al. [25] have shown that the function of *hgbA *as Hb receptor of *A. pleuropneumoniae *cannot be replaced. Still, considering the above mentioned findings, it is tempting to speculate that the *hpuB *genes are of some importance to iron regulation and constitute potential virulence factors of *A. pleuropneumoniae*. Recent observations support these assumptions. Along with a number of iron acquisition-associated genes of documented importance for virulence, e.g. *tonB1 *and *tonB2*, the *hpuB *gene cluster has been shown to be regulated by the global regulator 'fumerate and nitrate reduction regulator' (FNR) homologue, *hlyX *[46]. Furthermore, a study by Auger et al. [47] indicated some role of APL_1955 during infection. In a cell infection model, the authors found this gene to be up-regulated in *A. pleuropneumoniae *adhering to porcine lung cells when compared to non-attached (planktonic) bacteria from the same media [47]. Further research is needed to clarify the importance of the *hpuB *gene cluster.

Situated just next to the *hpuB *genes, but transcribed in the opposite direction were the ORFs APP_1957 and APP_1956. Both genes are of unknown function. APP_1957 displayed a putative lipoprotein_5 containing domain. APP_1956 was homologous to the carboxylase alpha subunit of N-methylhyantoinase B/acetone. Like Deslandes et al. [26], we observed these genes to be both highly up-regulated in response to iron deprivation in all serotypes. In fact, with log_2 _ratios of 3.36 and 2.74, respectively, they were the two most highly up-regulated genes of this study (Table 1).

#### Known and putative siderophores of *A. pleuropneumoniae*

The high affinity of OM receptors for their corresponding siderophore complexes, allow bacteria to more efficiently scavenge ferri-siderophores from their surroundings [19]. *A. pleuropneumoniae *is able to use exogenous hydroxamate and catecholate siderophores for promotion of growth [48]. Presently, only one siderophore receptor, the ferric hydroxamate specific, *fhuA*, has been described for this microorganism [31]. Besides the *fhu-*system, genome comparisons among virulent and commensal species of *Pasteurellaceae *indicate the presence of a second siderophore uptake system in *A. pleuropneumoniae *[41]. This possible ABC-type cobalamin/F^3+ ^siderophore operon consists of a periplasmic component (APL_0714) and an ATPase component (APL_0717) homologous to the siderophore binding protein fec*B*, and the ATP-binding protein, *fecE*, of *P. multocida*, respectively. The two permease components (APL_0715 and APL_0716), constitute the trans-membrane subunits of this putative siderophore transport system [41]. We observed an increased expression of APL_0714 to APL_0716 in response to iron deprivation in a seemingly co-regulated manner (Table 1). Under similar conditions, APL_0714 and APL_0717 were up-regulated in serotype 1 [26]. The increased expression during iron deprivation and the close homology of APL_0714 to APL_0717 with siderophore family transporters of other *Pasteurellaceae *support the hypothesis of a second siderophore acquisition system in *A. pleuropneumoniae*.

We also identified a third putative *A. pleuropneumoniae *siderophore receptor, ORF APL_0565, which was transcriptionally active under iron limited growth conditions in all six serotypes. This hypothetical *tonB*-dependent, ABC transporter ATP-binding protein displays 83%, homology with the *cirA *genes of *Actinobacillus minor *and shares 49% identity with the putative OM ferric siderophore of *N. meningtidis*. The estimated molecular weight of the *A. pleuropneumoniae cirA *protein, 72.6 kDa, is close to the 74 kDa size of the orthologous protein in *Escherichia coli*. In *E. coli*, the *cirA *gene was one out of six OM proteins identified as Fe^3+ ^siderophore receptors regulated by *fur *and iron concentrations [49-51]. One of the most effective ferric iron chelating compounds known is enterobactin, the chatecholate type siderophore of *E. coli *and several other bacteria [52]. As previously mentioned, *A. pleuropneumoniae *is able to use catecholate siderophores for promotion of growth [48]. Possibly the *fec*-like operon and the hypothetical *cirA *protein encode the siderophore receptors responsible for uptake of this iron-chelator in *A. pleuropneumoniae*. Contrary to the *fhu*-system, whose regulation is iron-independent [32], the putative catecholate receptors appear to be iron-repressible.

#### Other iron uptake systems

The iron and *fur *repressed operons *ycdNOB *of *E. coli *and *ywbLMN *of *Bacillus subtillis *are predicted elemental iron uptake systems orthologous to the copper oxidase-dependent Fe(III) uptake system, *Ftrp*/*Fet3p*, of *Saccharomyces cerevisiae *[53]. In *B. subtillis*, *ywbLMN *appears to be involved in the uptake of free ferric iron [54]. An *ycdNOB*-*ywbLMN*-like *fur *repressed iron-transport system may also be present in *A. pleuropneumoniae*, represented by the ORFs APL_0670, APL_0669, and APL_0668. All three ORFs were up-regulated in *A. pleuropneumoniae *serotype 1 under iron limitation [26]. We found only the last two ORFs to be up-regulated in all the included serotypes under similar growth conditions. APL_0669 encoded a putative peroxidase *YwbN *precursor. APL_0668, a putative periplasmic lipoprotein involved in iron transport, is 58% identical to the *ycdO *of *Gemella haemolysans*. By analogy of the well-characterized *Saccharomyces cerevisiae Ftrp*/*Fet3p *system, it has been suggested that *ywbLMN *functions as a Fe(III) permease [54]. In *B. subtilis*, the *ywbLMN *iron uptake system was required for growth in iron-limited medium lacking citrate, and in *E. coli *the *ycdNOB *proteins function as an additional ferrous iron-uptake systems [54,55]. The functional role of the putative operon, *ywbLMN*, of *A. pleuropneumoniae *is presently unknown.

#### Other up-regulated genes

Also up-regulated in the iron depleted bacteria was a potential co-regulated operon consisting of the copper-transporting P-type ATPase, *copA *gene (APL_1265), a putative cation transport ATPase (APL_1264), and a predicted metal-binding protein (APL_1263). In eukaryotes, several interdependent connections between copper and iron homeostasis have been described. Knowledge of such processes in bacterial systems is still limited [56]. In *E. coli*, 2,2'-dipyridyl supposedly only effects iron and not the copper and magnesium levels of the bacterial cell [57]. Still, we have reason to believe that the up-regulation of at least the *copA *in serotype 6 may be an artifact introduced by this high affinity chelator. Primarily because the qPCR analysis, testing the possible effect of 2,2'-dipyridyl, indicated that this regulatory event was un-related to iron deficiency. Secondly, in a similar study, the *copA *gene of *Staphylococcus aureus *was expressed in the media but not *in vivo *[58]. Finally, none of the above mentioned three genes were up-regulated in *A. pleuropneumoniae *serotype 1 when EDDHA was used to obtain iron restricted conditions [26].

We also saw an up-regulation of the Fe/S biogenesis protein, *nfuA*. In *E. coli*, *nfuA *is required for maturing Fe/S proteins under oxidative stress and iron starvation [59]. Overall, observations from studies involving *E. coli *and other bacteria seem to point towards a general role for the ubiquitous bacterial protein *nfuA *in Fe/S protein folding under stress conditions [59].

Some of the up-regulated genes of the microarray were not directly related to iron-homeostasis, but were situated next to known or putative iron regulators and displayed similar expression ratios. The position and the seeming co-regulation of these ORFs indicate some indirect involvement in iron homeostasis. For example, three loci immediately downstream of the *tbpA1 *gene, APL_1566 to APL_1564, were all transcribed in the same direction and up-regulated to a similar extend as the *tbpBA *genes (Table 1). The *xylB1 *gene (APL_1564), encoding pentolose and hexulose kinase, was also up-regulated in *A. pleuropneumoniae *serotype 1 and *M. haemolytica *under iron-deprivation [26,29].

The fumerate and nitrate reduction regulator (FNR) homologue, *hlyX *has a global regulatory role in *A. pleuropneumoniae *where it induces the expression of genes involved in anaerobic metabolism and simultaneously represses genes involved in aerobic metabolism [46]. Also the expression of the iron acquisition-associated genes, *frpB*, the *tonB1 *and *tonB2*, and the newly described putative *hpuB *cluster were found to be positively regulated by *hlyX *under anaerobic conditions [46]. In this and previous studies of *Pasteurellaceae *[26,27], the gene regulations under iron restricted condition were not completely in accordance with the expected FNR regulation. In *A. pleuropneumoniae *and *P. multocida*, the *hlyX *gene was up-regulated while genes that are normally transcriptionally activated by *hlyX *were down-regulated (see below). *HlyX *is important but not essential to virulence and other not-yet-identified regulators may be involved in gene regulation under anaerobic conditions [60].

### Genes down-regulated under iron restriction

In agreement with observations in *A. pleuropneumoniae *serotype 1, *P. multocida*, and *M. haemolytica *[26,27,29], more than half of the 67 down-regulated genes under iron shortage belonged to the COG group "energy production and conversion". The repressed genes were the main cellular iron consumers, mostly genes coding for metabolic enzymes dependent on Fe-S clusters or other iron cofactors e.g. hydroxylamine reductase, cytochrome c peroxidase, and the fumerate reductase operon (Table 2). As for deducting any hypothesis regarding difference in virulence among serotypes, the genes which displayed significant serotype related down-regulation in response to iron limitation were not very useful (genes marked with a number sign in Table 2). For all the 9 genes, a low virulent (serotype 3) and a high virulent (serotype 5) serotype seemed to be the least repressed.

Many down-regulated genes coded for proteins involved in anaerobic metabolism, some of which are known to be regulated by one or both of the two important anaerobic regulators, *hlyX *and anoxic redox control protein A (*arcA*) [46,61]. Genes/operons down-regulated in this study and expected to be controlled by *hlyX *were: 1) terminal reductases, transferring respiratory chain electrons to alternative electrons acceptors: dimethyl sulfoxide reductase (*dmsBA*), periplasmic nitrate reductase (*napFDAGHBC*), and the TMAO reductase (*torYZ*), 2) genes encoding enzymes involved in the oxidation of high-energy substrates e.g. the Ni/Fe cofactor dependent hydrogenases (*hyaAB *and *hybAB*).

Although we observed no up-regulation of *arcA*, a number of genes know to be controlled by *arcA *were also differentially expressed in this study [61]. Genes negatively regulated by *arcA *and likewise down-regulated in this study were: the OM protein precursor (*ompW*), the formate dehydrogenase genes (*fdnI *and *fdxHG*), the putative dehydrogenase subunit (*ykgE*), the putative Fe-S electron transport protein (*ykgF*), and the nitrate reductase operon (*nrfDBA*). As with the *hlyX *regulator, the expression pattern of some genes did not concur with the expected pattern of *arcA *regulation [61]. For example, the genes encoding L-lactate dehydrogenase (*lldD*) and a putative oxygen-independent coproporphyrinogen III oxidase (*chuW*), both expected to be depressed by *arcA*, were up-regulated under iron-deprivation in *A. pleuropneumoniae*. Likewise, a number of genes, the anaerobic dimethyl sulfoxide chain precursors (*dmsAB*), the serine dehydratase/transporter protein genes (*sdaAC*), and a putative effector of murein hydrolase (*lrfA*) under positive regulation of *arcA*, were all down-regulated in this experiment.

Clearly *hlyX *and *arcA *were not the main regulators in *A. pleuropneumoniae *under iron starvation. Besides the discrepancies just described, far from all the down-regulated metabolic genes, e.g. fumarate reductase genes (*frdABCD*) and the Na+-translocating NADH-ubiquinone oxireductase subunits (*nqrFEDCBA*) have been proven to be regulated by *hlyX *and *arcA *[61]. As previously mentioned, an essential role of ryhB in iron metabolism has been demonstrated in *E. coli*, *Vibrio cholera *and a number of other bacteria [62]. The ubiquitous RNA-binding protein, Hfq is a key factor in regulations by sRNAs in bacteria [35]. In *E. coli *ryhB sRNA acts as an Hfq-dependent regulator of the acquisition and storage of iron [63]. Although not yet proven for *A. pleuropneumoniae *or other *Pasteurellaceae*, the recent discovery of Hfq homologues in *A. pleuropneumoniae*, *P. multocida*, and *H. influenza *makes it more likely that sRNAs participate in controlling iron metabolism in members of this family [35]. Furthermore, the overall down-regulatory response to iron limitation, observed in this and previous studies in *Pasteurellaceae*, fits well with the concerted actions of *fur *and rhyB described in *E. coli *[63]. *Fur *inactivation enables the expression of the sRNA regulator, ryhB, which then limits the usage of iron to crucial proteins by repression of as many non-essential iron-using proteins as possible [20].

## Conclusions

By studying the intra-species variation in the transcriptional response of *A. pleuropneumoniae *to iron limitation, we identified a set of core genes important to the iron adaptive response of this organism. Not surprisingly, these included known virulence factors such as the *tonB*-system, *tbpBA *and *hgbA*, but also a number of newly described potential iron acquisition genes previously identified in *A. pleuropneumoniae *serotype 1, e.g. the putative hemoglobin-haptoglobin binding proteins and the *yfe*-system [26]. We confirmed the up-regulation of the latter genes in an additional five serotypes of *A. pleuropneumoniae *under iron limitation. Not previously observed in *A. pleuropneumoniae *was the up-regulation of a putative siderophore, *cirA*. The *hpuB *cluster proved to be interesting for several reasons. Firstly, all three genes displayed serotype specific expression under iron limitation. Of the six serotypes, serotype 3 was the only variant in which none of the three ORFs were significantly up-regulated under iron deprivation. Serotype 3 is also considered to be one of the least virulent serotypes of *A. pleuropneumoniae*. Secondly, in ORF APL_1953 a variable region was identified which differentiated serotypes 2, 5 and 6 from serotypes 1, 3 and 7. The gene was expressed in all serotypes. Except for serotype 3, we could not directly correlate the observed variances in the *hpuB *ORFs with serotype related differences in pathogenesis. Still, it would be interesting to study further the implications of these divergences in gene expression and length and to determine the role the *hpuB *gene complex concerning iron acquisition and virulence in *A. pleuropneumoniae*. In all, the combined effect of iron-depletion and serotype proved to be modest, indicating that serotypes of both medium and high virulence at least *in vitro *are reacting almost identical to iron restriction. This is perhaps not very surprising, considering the functional importance of the core genes involved in iron regulation.

Attesting to the quality of the array were: 1) the concordance of gene regulation within the operons, 2) the agreement of results between this and previous transcriptional studies of *Pasteurellaceae *under conditions of iron limitation [26-29], and 3) the high correlation between the microarray and qPCR expression ratios. Consequently, the results attest the utility of this novel pan-genomic *A. pleuropneumoniae *microarray. The design of this array is publicly available and will hopefully be applied for multiple purposes of serotype comparisons in the future.

## Methods

### Bacterial strains and growth conditions

The following strains of *A. pleuropneumoniae *were used in this investigation: Serotype 1 (4074), serotype 2 (4226), serotype 3 (1421), serotype 5b (L20), serotype 6 (7712640), serotype 7 (WF87). For the iron depletion study, an overnight culture was diluted 1:10 in brain heart infusion broth supplemented with 0.03% NAD (Sigma-Aldrich, Copenhagen, Denmark) and incubated until the optical density at 660 nm was 0.6, representing the exponential growth phase. The culture was split into two 10-ml portions. Iron-depleted conditions were established in one of the flasks by addition of 300 μM 2,2'-dipyridyl (Sigma-Aldrich). For serotype 2 and 6, iron repletion experiments were also performed: to a third 2,2'-dipyridyl containing flask, 300 μM ammonium iron(II) sulfate hexahydrate (Sigma-Aldrich) was added. Next, the cultures were grown under shaking at 37°C for 30 min, after which one volume of RNA*later *stabilization reagent (Ambion, Cambridgeshire, United Kingdom) was added to the cultures. Samples were harvested by centrifugation, immediately resuspended in one volume of PBS and 5 volumes of RNA*later*. Samples were stored at 4°C until extraction. For each serotype, four experiments were performed on different days.

### RNA isolation and reverse transcription

Total RNA was isolated from 1.5-ml portions of bacterial samples by using an RNeasy mini kit (QIAGEN, Hilden, Germany) as described by the manufacturer. Genomic DNA was eliminated by RNase-free DNase I treatment during the isolation procedure. After RNA extraction, the material was further treated by TURBO™ DNase, according to the protocol provided by the manufacturer (Ambion). The RNA concentration and quality were measured by NanoDrop (Thermo Scientific, Wilmington, DE, USA) and Agilent 2100 Bioanalyzer (Agilent Technologies, Santa Clara, CA, USA), respectively. Quality requirements were: A260/A280 ≥ 1.8 and A260/A230 ≥ 1.8 and RIN ≥ 7.5. Samples not meeting this standard were discarded and new extractions performed. Exceptions to this rule were serotype 5, where the RIN numbers consistently remained between 5.5 and 6, and serotype 6, generally displaying RIN numbers just below 7.

### Designing the custom array

The arrays used in this project were based on the NimbleGen 12-plex platform, officially released in a news statement on Nov. 19, 2008. The custom probe set for the arrays was build around a set of 7 core genomes representing all publically available *A. pleuropneumoniae *and *Actinobacillus succinogenes *genomes in GenBank and RefSeq [PMID: 18073190]. These included *A. pleuropneumoniae *serotype 1 str. 4074 [GenBank: AACK00000000], serotype 2 str. 4226 [GenBank: ADXN00000000], serotype 3 str. JL03 [GenBank: NC 010278], serotype 5b str. L20 [GenBank: NC 009053], serotype 6 str. Femo [GenBank: ADXO00000000], and serotype 7 str. AP76 [GenBank: NC 010939]. [64-66]. In total, 15018 target genes were identified from these data.

### Constructing the Target Gene Set

All genes were aligned all-against-all and any two genes sharing more than 90% identity over the full length of the shortest sequence were connected together into gene networks. Representatives from each gene network were selected using the algorithm of Hobohm et al. [67] for maximizing the size of the selected set. This algorithm eliminates redundancy in a network through the removal in order of the most highly connected members. This produced a set of 4876 *Actinobacillus *target genes. In total, each array consisted of 130.194 active probes excluding NimbleGen control probes. Each gene was covered by an average of 26.7 probes. The design of this array is publicly available at NimbleGen (091013_DTU_Actino_xRNA).

### Probe Selection for Target Genes

Probes for the target gene set were selected using the OligoWiz program [68,69] applying the following weighting of the scores: Cross-Hybridization: 39.0%, Delta Tm: 26.0%, Folding: 13.0%, Position: 13.0%, Low-complexity: 9.1%. The probe length was adjusted between 44 and 52 bp with an average of 48 bp to ensure comparable hybridization strengths. Because OligoWiz was originally designed for use with single genomes in mind, it was necessary to modify the program, specifically the mechanisms screening for cross-hybridisation which needed to be less strict. A new scheme was devised by introducing a log-transformation in the underlying calculations. The net effect is negligible near the upper boundary of the score, but near the lower boundary it increases the discriminatory power of the method.

### Preparation of labeled double-stranded DNA

Ten micrograms of total RNA from each sample was reverse transcribed using SuperScript II (Invitrogen, Carlsbad, CA) and Random Hexamer Primers (Invitrogen) according to the NimbleGen Arrays User's Guide (Gene Expression Analysis v3.2). The generated cDNA was incubated with 1 μl of 4 mg/ml RNase A solution (Promega Corporation, Madison, WI, USA) at 37°C for 10 min, and then phenol-chloroform extracted. Samples were centrifuged in Phase Lock Gel Tubes (5 Prime, Hamburg, Germany) at 12,000 × g for 5 minutes and precipitated with 80% ethanol. Pellets were air dried in a SpeedVac and rehydrated in 20 μl of ultrapure water (Ambion). Finally the samples were measured by NanoDrop to ensure that the cDNA met the following quality requirements: A260/A280 ≥ 1.8 and A260/A230 ≥ 1.8. NimbleGen One-Color DNA Labeling kit (NimbleGen Systems, Madison, WI) was used for Cy3 labeling of cDNA samples according to the NimbleGen Arrays User's Guide. Briefly, 1 μg double-stranded cDNA was incubated for 10 min at 98°C with Cy3-random Nonamers and then quick-chilled in a ice-water bath for 10 min. The addition of 100 mM of deoxynucleoside triphosphates and 100 U of Klenow fragment (New England Biolabs, Ipswich, MA) was followed by incubation at 37°C for 2 h. The reaction was stopped by adding 0.1 volumes of 0.5 M EDTA, and the labeled cDNA was precipitated with isopropanol.

### Hybridization and analysis of arrays

A hybridization kit (NimbleGen Systems, Madison, WI) was used for the hybridization step. Cy3-labeled samples were resuspended in the recommended amount of hybridization buffer and denatured at 95°C for 5 min. Slides were placed in HX12 NimbleGen Mixer and 6 μl of sample loaded though the fill port. Hybridization was performed for 20 h at 42°C (NimbleGen Hybridization System 16). The arrays were washed using a wash buffer kit (NimbleGen Systems), dried in a microarray dryer (NimbleGen), and scanned at a 5 μm resolution using the NimbleGens MS 200 scanner (NimbleGen Systems).

### Quantitative real-time PCR

Gene quantification was performed with a Rotor-Gene 6000 (Corbett Research, Sydney, Australia). The primers were designed using Primer3 (v. 0.4.0) [70]. The sequences of the primers are listed in Table 3. Each PCR was performed in a 25 μl reaction mixture containing 12.5 μl QuantiTect SYBR Green PCR master mix (Qiagen, Hilden, Germany), a primer concentration of 0.3 μM and 7 ng of cDNA. Three biological replicas were included for each sample. The thermal cycling conditions were as follows: 15 min at 95°C, followed by 40 cycles of 15 s at 94°C, 20 s at 55°C, and 20 s at 72°C. Data collection was performed during each extension phase. Positive controls (DNA), a negative control (distilled water), and RT-negative controls (total RNA sample) were included in each run. Melting curve analysis was performed, which for all primer sets resulted in single product-specific melting curves.

**Table 3:** List of primers used for quantitative real-time PCR and sequencing

Target gene	Forward primer	Reverse primer
**glyA**	CAAGCGAATGCAGCTGTTTA	CTGTGATGCCGTAGAGGACA
**pykA**	GTACGGATGCGGTAATGCTT	GTACGGATGCGGTAATGCTT
**tpiA**	CTACGAACCGATTTGGGCTA	CCGCCGTATTGGATAATCAC
**exbB1**	CCGGAATTGCGACAAATAGT	CCGTTCATTGGGTTATTTGG
**tonB1**	CATTGCATTGCCATAACCAG	AAAAGCGCCTGAAAAGATCA
**tbpA1**	AGGAATGACGTTGGTTTTGC	ATTGCAGGTAGGGCTGATTG
**copA**	CTATGAAGCCAGCGTGATGA	CCAAATAGAACCGCTTTCCA
**tonB2**	GCCTTGTACCGCATTAGGAA	CTCAGCCTAAGCCGAAAGAA
**hlyX**	TTTTACGTTGAGCGAACACG	ACGCCGTAATTTGTTCTTCG
**cirA**	TACGCTCTCCGGTGTGTATG	GTTGCGGTAGAAGCACCTTT
**ywbN**	TCGCAAATGGGCTTTAATTC	CTTTCAGCCAACCGTCTTGT
**hpuB (APL_1953)**	AACATCGTGTAAGCGCCTCT	GCCCTCATCATCGGTATCAC
**hpuB (APL_1954)**	ACGAAATGTTCTCCGGTACG	GATAGCCGGTCGAAACGTAA
**hpuB (APL_1955)**	GGTTCGGCAACCTTATTTGA	CGTTCTAACCCGCGTAATTC
**lldD**	AATGCCCTTGATTACCATCG	GTAAACCGCATACGCTTGGT
**hyaA**	TTTACCGGGTATGCCGATTA	GTGTCCTTCATCGCCGTATT
**hybB**	TAATACCGGCAAAGGCTGTC	ACTTTCGCAAACTCGCCTAA
**napB**	ACCCGTCGTGCTTGATACTT	GGCTTATACCAACCCGCATA
**nqrC**	CCGTAGCTAAAGGTGCTTCG	TTAGCTCCCATTGCTGCTTT
**fdxG**	TACTGTTCTGTCGGCTGTGG	GACTTACCGGATGGTCAGGA
**ykgE**	GTTTAAACGACCGGCAATGT	AACAAACCTGTTGCGGTTTC
hpuB (APL_1954)*	TCGGAGGAAAACTCGCTTTA	TAACCACCGGTCGGAAAATA

All primers were designed by Primer3 http://frodo.wi.mit.edu/primer3/ and purchased at DNA Technology A/S, Risskov, Denmark. *Primers used for sequencing.

### Relative quantification

The Excel-based relative expression software tool, REST 2009 (V2.0.13), was applied for group wise comparison and statistical analysis of the qPCR data http://rest.gene-quantification.info/[71]. The relative expression ratios were calculated by a mathematical model, which included an efficiency correction for real-time PCR efficiency of the individual transcripts [72], as follows:

$$\text{Ratio}={\left(E_{\text{target}}\right)}^{\Delta \text{CP}}{{}_{\text{target}}}^{\left(control-sample\right)}/{\left(E_{\text{ref}}\right)}^{\Delta \text{CP}}{{}_{\text{ref}}}^{\left(control-sample\right)}$$

The relative expression ratio of a target gene was computed based on its real-time PCR efficiencies (*E*) and the crossing point difference (ΔCP) for an unknown sample versus a control. For each gene, cDNA dilution curves were generated and used to calculate the individual real-time PCR efficiencies (*E *= 10^[-1/slope]^). The geometric mean of the three internal reference genes was used to correct the raw values for the genes of interest (Table 4).

**Table 4:** Relative expression results from REST analysis of qPCR data

**No**.	Gene	Type	Reaction Efficiency	Expression	Std. Error	P(H1)*
	*glyA*	Reference	0.91	1.33		
	*pykA*	Reference	0.99	1.00		
	*tpiA*	Reference	0.99	0.75		
**1**	*exbB1*	Sample	1.00	13.94	4.26 - 35.88	0.000
**2**	*APL_1953*	Sample	0.99	6.82	2.45 - 20.84	0.000
**3**	*APL_1954*	Sample	0.98	5.09	1.49 - 16.29	0.000
**4**	*APL_1955*	Sample	1.00	6.68	2.44 - 20.64	0.000
**5**	*cirA*	Sample	0.85	2.26	0.92 - 5.30	0.001
**6**	*ywbN*	Sample	1.00	3.76	1.87 - 7.70	0.000
**7**	*copA*	Sample	1.00	6.98	2.79 - 17.86	0.000
**8**	*hlyX*	Sample	0.95	1.78	0.70 - 5.14	0.017
**9**	*tbpA1*	Sample	0.90	7.82	2.62 - 18.50	0.000
**10**	*tonB1*	Sample	0.94	12.60	3.56 - 34.29	0.000
**11**	*tonB2*	Sample	1.00	1.65	0.75 - 3.66	0.012
**12**	*lldD*	Sample	0.98	15.32	1.69 - 90.73	0.000
**13**	*napB*	Sample	0.94	0.18	0.07 - 0.58	0.000
**14**	*nqrC*	Sample	0.93	0.36	0.18 - 0.74	0.000
**15**	*ykgE*	Sample	0.89	0.12	0.04 - 0.45	0.000
**16**	*hyaA*	Sample	1.00	0.05	0.02 - 0.21	0.000
**17**	*fdxG*	Sample	1.00	0.30	0.07 - 1.37	0.004
**18**	*hybB*	Sample	0.93	0.09	0.03 - 0.37	0.000

*The hypothesis test represents the probability of the alternative hypothesis that the difference between the sample and control groups is due only to chance. Results are based on 6000 randomizations.

### Data analysis of Microarray data

The data discussed in this publication have been deposited in NCBI's Gene Expression Omnibus [73] and are accessible through GEO Series accession number GSE24470 http://www.ncbi.nlm.nih.gov/geo/query/acc.cgi?acc=GSE24470. Data analysis of the microarrays was performed in "RGui" version 2.9.2 (2009-08-24) http://cran.r-project.org/bin/windows/base/, using the package "Oligo". The Robust Multichip Average function was applied for normalization of the microarray data [74]. By this method, the expression measure is given in log_2 _base. Mean log_2 _expression values of the four biological replicates are given in Table 1 and 2. Two-way analysis of variance (ANOVA) was used to test the effect of treatment (F1: bacterial response to iron deficiency versus control) and serotype (F2 response between serotypes 1, 2, 3, 5, 6 and 7) and the combined effect of treatment and serotype (F1:F2).

### Construction of genomic atlases

The program BLASTatlas http://www.cbs.dtu.dk/ws/BLASTatlas, was used for mapping and visualizing whole genome homology of expressed genes [75]. Using the published genome of *A. pleuropneumoniae *serotype 3 str. JL03 as a reference strain, the expression values of control cultures and iron depleted cultures of the six serotypes were compared to each other.

## Abbreviations

OM: outer membrane, Hb: haemoglobin, sRNA: small non-coding RNA, *Fur*: ferric-uptake regulator protein, COGs: Clusters of Orthologous Groups, qPCR: Real-time quantitative RT-PCR, FNR: Fumerate and Nitrate reduction Regulator, *ArcA*: Anoxic redox control protein A.

## Authors' contributions

All authors participated in conceiving and designing the study. KK performed growth studies, microarray and qPCR analysis and drafted the manuscript. CF designed the pan-genomic microarray and helped with the downstream data analysis and manuscript. MB, CF and ØA revised the manuscript. All authors read and approved the final manuscript.

## Supplementary Material

Additional file 1 **Figure S1. The effect of 2,2'-dipyridyl with and without the addition of exogenous iron**. Results of qPCR expression analysis of *A. pleuropneumoniae *serotype 2 (A) and serotype 6 (B) grown in media with 300 μM of 2,2'**-**dipyridyl only (dark blue bars) or with 300 μM of 2,2'**-**dipyridyl and 300 μM of ammonium iron(II) sulphate hexahydrate (light blue bars). Click here for file
