# Supplementary figures and images for: Comparative profiling of the transcriptional response to iron restriction in six serotypes of Actinobacillus pleuropneumoniae with different virulence potential
Source: BMC Genomics. 2010 Dec 9;11:698. doi: 10.1186/1471-2164-11-698 (PMC3091793; doi:10.1186/1471-2164-11-698)

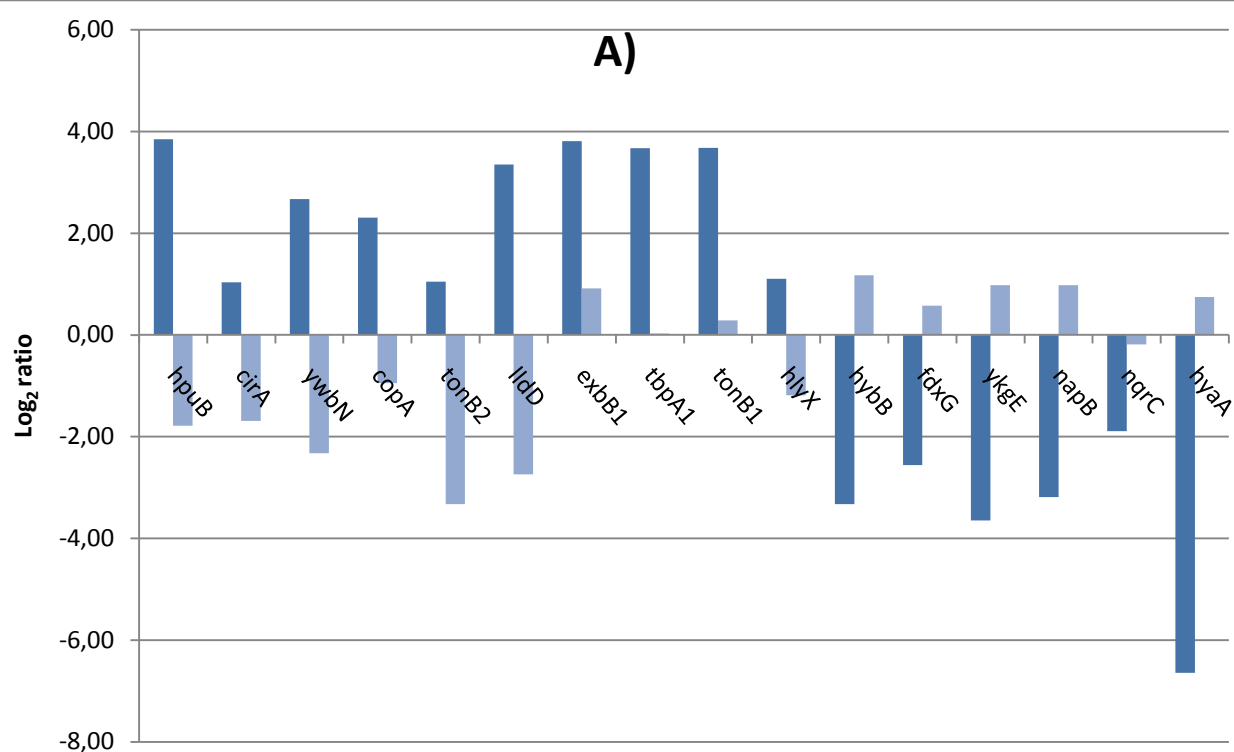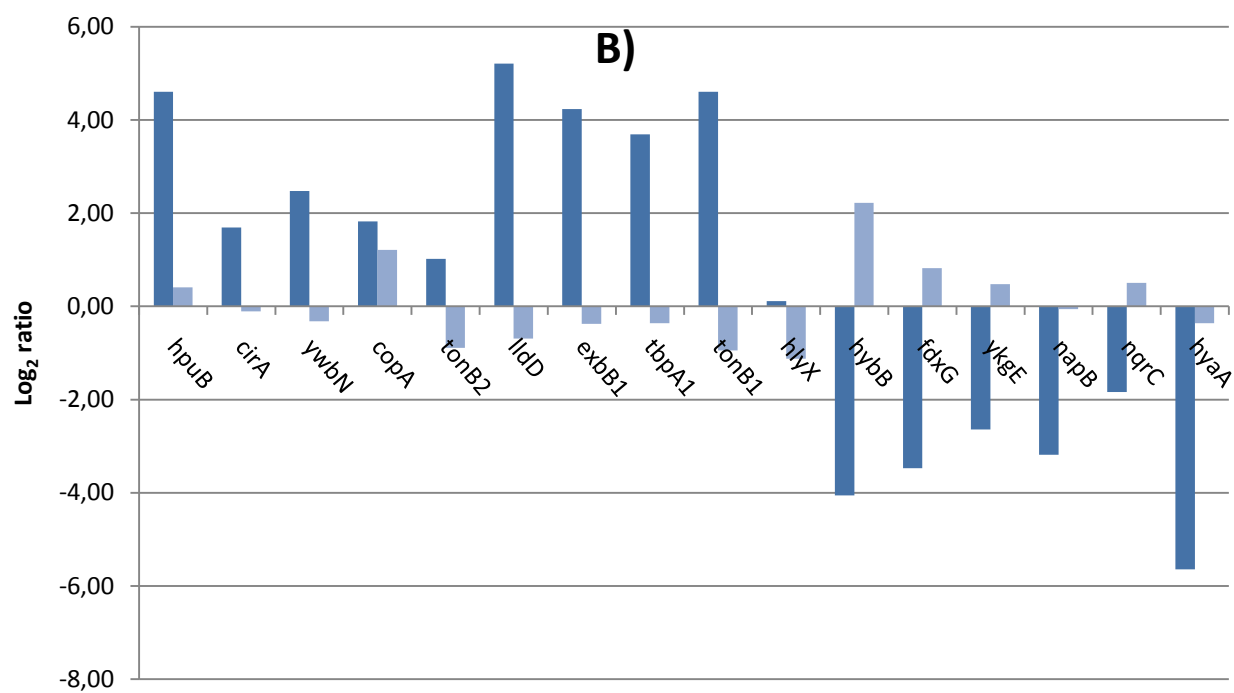

Supplement: Additional file 1 — Figure S1. The effect of 2,2'-dipyridyl with and without the addition of exogenous iron. Results of qPCR expression analysis of A. pleuropneumoniae serotype 2 (A) and serotype 6 (B) grown in media with 300 μM of 2,2'-dipyridyl only (dark blue bars) or with 300 μM of 2,2'-dipyridyl and 300 μM of ammonium iron(II) sulphate hexahydrate (light blue bars). [file 1471-2164-11-698-S1.PDF]
